# Radiological Approach to Assessment of Lower-Limb Alignment—Coronal and Transverse Plane Analysis

**DOI:** 10.3390/jcm13226975

**Published:** 2024-11-19

**Authors:** Anna Michalska-Foryszewska, Piotr Modzelewski, Katarzyna Sklinda, Bartosz Mruk, Jerzy Walecki

**Affiliations:** 1 Radiological Diagnostics Center, The National Institute of Medicine of the Ministry of Interior and Administration, 02-507 Warsaw, Poland; 2 Clinic of Orthopedics and Traumatology, The National Institute of Medicine of the Ministry of Interior and Administration, 02-507 Warsaw, Poland

**Keywords:** lower-limb alignment, X-ray imaging, osteotomy, computed tomography, rotational deformities

## Abstract

Lower-limb alignment deformities constitute a significant clinical concern, as they can lead to serious complications, including progressive degenerative diseases and disabilities. Rotational deformities may give rise to conditions such as joint arthrosis, patellar instability, and the degeneration of the patellofemoral cartilage. Therefore, a comprehensive evaluation of lower-limb alignment is essential for the effective patient management, preoperative planning, and successful correction of these deformities. The primary assessment method employs full-length standing radiographs in the anteroposterior (AP) projection, which facilitates accurate measurements of the anatomical and mechanical axes of the lower limb, including angles and deviations. The outcomes of this analysis are vital for the meticulous planning of osteotomy and total knee arthroplasty (TKA). In addition, computed tomography (CT) provides a specialized approach for the precise evaluation of femoral and tibial rotation. In this area, there are potential opportunities for the implementation of AI-based automated measurement systems.

## 1. Introduction

Lower-extremity alignment abnormalities represent a prevalent issue with multifaceted etiology, which may occur from congenital anomalies, metabolic irregularities, or post-traumatic distortions [1]. The evaluation of lower-extremity alignment often relies on conventional radiographic techniques, predominantly utilizing full-length standing radiographs in the anteroposterior (AP) plane to present the anatomy of the lower limb from the femoral head to the ankle [2]. The advantage of radiographs is that they are widely available, easily accessible, and relatively inexpensive [3]. This examination is useful in assessing the mechanical axis of the lower extremity. It also enables the measurement of the level of deformities and permits strategic preoperative planning that aims to correct the abnormalities. Furthermore, this approach is crucial in total knee arthroplasty (TKA) procedures since it guides decisions regarding optimal bone resections. It is also essential for the comprehensive assessment of postoperative outcomes [4,5]. Thus, recreating the normal alignment of the lower extremity plays a pivotal role in achieving a satisfactory outcome following long bone fractures and limb reconstruction [6,7]. Alternative modalities like computed tomography (CT) and magnetic resonance imaging (MRI) are pivotal for precise and detailed diagnostic insights in more complicated cases [8]. In this field, there are promising prospects for the implementation of AI-based automated measurement systems.

This article presents a comprehensive review of the current literature on the radiological assessment of lower-limb alignment, deformities, and advanced imaging and analytical capabilities enabled by artificial intelligence.

### 1.1. Proper Examination Techniques

The full-length standing radiograph in the AP projection serves as the primary modality for evaluating lower-limb alignment. This technique allows for the precise determination of the mechanical and anatomical axes of both the femur and tibia, as well as the measurement of angles of the lower limb. A crucial component of accurate assessment is the standardized positioning of the patient prior to the examination, which is essential for achieving consistent and reliable results. To facilitate an accurate evaluation of the X-ray image, it is imperative that the imaging is conducted in the AP projection with a horizontally aligned X-ray beam directed at the hip, knee, and ankle. Ensuring the proper alignment of the patella within the AP projection necessitates centering it between the femoral condyles. Conventionally, an 8–10° lateral rotation of the feet results in this alignment. However, certain conditions, such as torsional deformities, might induce the medial or lateral displacement of the patella. In the above-mentioned instances, the appropriate positioning could be attained by internally or externally rotating the lower leg until the patella is centralized between the femoral condyles. External rotation (ER) causes less apparent valgus and leads to more varus, and, conversely, internal rotation (IR) causes more valgus and leads to less varus [8,9].

### 1.2. Evaluation of Lower-Limb Alignment

**Figure 1:**
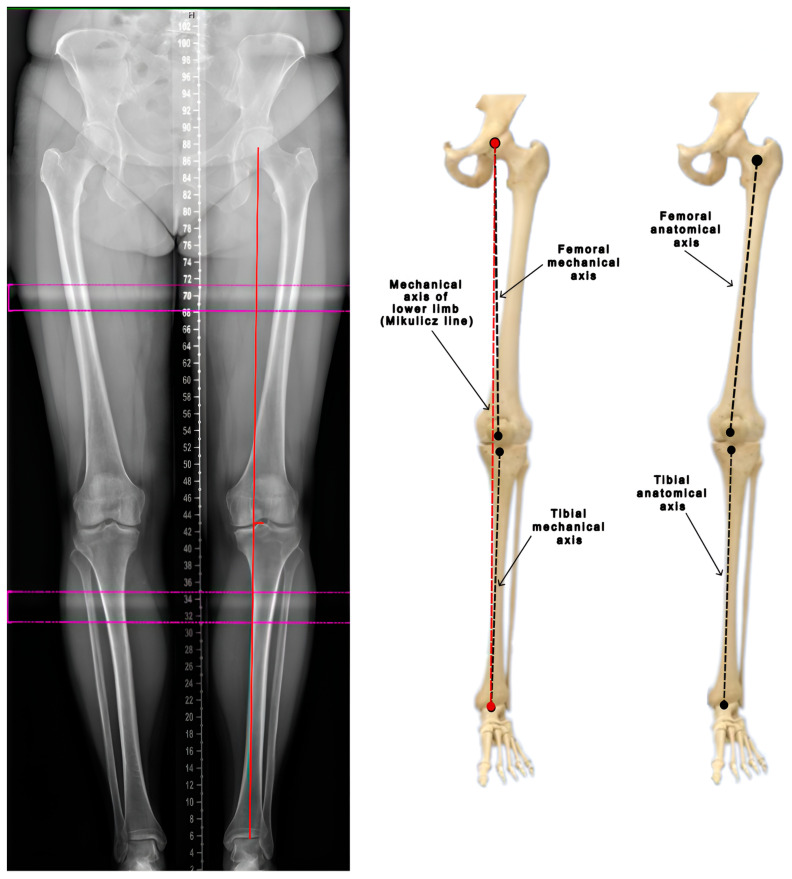
Evaluation of the mechanical and anatomical axes of the lower limb (based on Luís et al. [8]).

The anatomical axes of the femur and tibia align with the mid-diaphyseal line of each respective bone. The mechanical axis of the femur is defined by a line extending from the center of the femoral head to the center of the femoral intercondylar notch. Similarly, the mechanical axis of the tibia is delineated from the interspinous groove to the center of the talus or tibial plafond. Consequently, in the tibia, both axes are physiologically similar to each other. An additional mechanical axis of the lower limb, known as the Mikulicz line, is defined by a line that extends from the center of the femoral head to the midpoint of the tibial plafond (the center point of the ankle joint) (Figure 1). The lateral or medial deviation of this axis from the center of the knee joint is measured in millimeters, and this range is referred to as the mechanical axis deviation (MAD). In the correct physiological position, the Mikulicz line should run approximately 4 ± 2 mm medial to the center of the knee. The line’s course is indicative of specific alignment issues: a lateral deviation suggests valgus alignment, while a more medial deviation indicates varus alignment. The joint line convergence angle (JLCA) is formed by a line that touches the femoral condyles and a line that touches the tibial plateau. Under normal conditions, these lines are nearly parallel, showing a slight medial convergence of 0–1 degrees. In valgus knees, the JLCA opens medially, while in varus knees, it opens laterally [8,9,10].

### 1.3. Evaluation of Lower-Limb Angles

**Figure 2:**
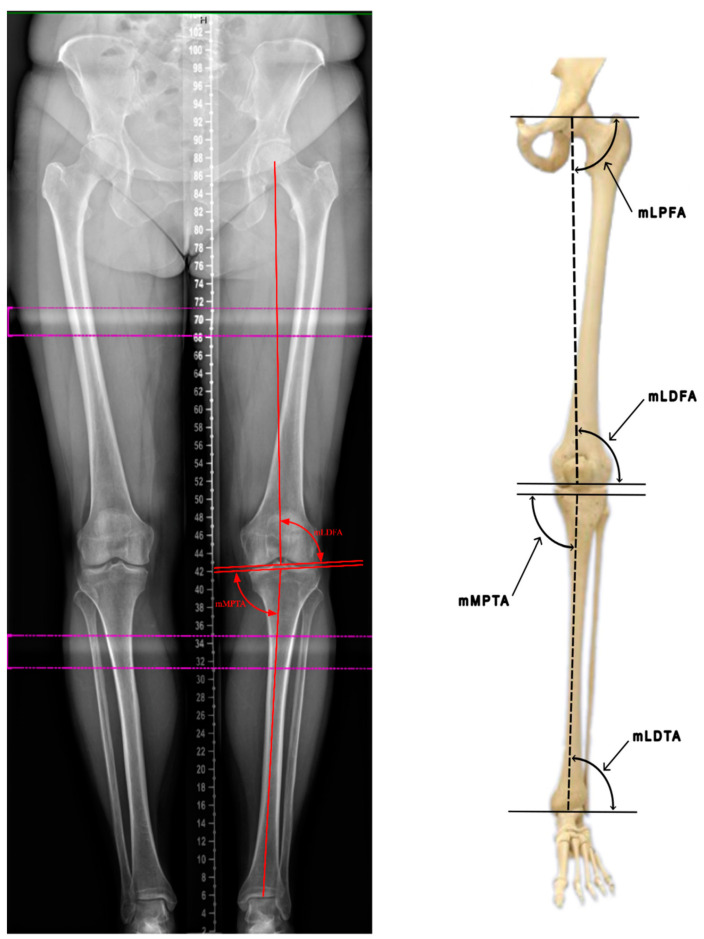
Evaluation of lower-limb angles (based on Luís et al. [8]).

The anatomical tibiofemoral angle (TFA), also known as the hip–knee–ankle angle (HKA), is measured between the anatomical axes of the femur and the tibia, with a normal range of 1–1.5° in healthy individuals. In order to accurately measure the TFA, a line corresponding to the anatomical axis of the femur should be extended to form an angle with the anatomical axis of the tibia. The mechanical axis of the femur forms a physiological angle of 6° ± 1° with the anatomical femoral axis, referred to as the anatomical–mechanical femoral angle (MFA) (Figure 2).

Specific angle measurements based on the mechanical axis of the lower-limb bones and their physiological ranges are shown in Table 1.

The femoral torsion angle is measured between the axis of the femoral head and neck in the transverse plane and a tangent line to the posterior femoral condyles. The normal range for this angle is 15.6° ± 6.7°.

The tibial torsion angle is determined by the angle between the tangent lines at the posterior margins of the proximal tibial condyles and the central distal transmalleolar axis. The physiological range for this angle is 23.5° ± 5.1° [8,10].

Abbreviations: mechanical lateral proximal femoral angle (mLPFA); mechanical lateral distal femoral angle (mLDFA); mechanical medial proximal tibial angle (mMPTA); mechanical lateral distal tibial angle (mLDTA).

### 1.4. Lower-Limb Deformities

Deformities in the alignment of the lower limb constitute a significant clinical issue, potentially leading to progressive degenerative joint diseases and disability. Varus and valgus deviations represent the most prevalent types of lower-limb deformities. These misalignments result in abnormal load distribution across the knee joint compartments, with varus deformities causing increased stress on the medial compartment and valgus deformities affecting the lateral compartment. Such imbalances contribute to chronic mechanical overload and accelerated cartilage degeneration. Osteotomy procedures, aimed at realigning the weight-bearing axis and redistributing mechanical loads on the affected limb, play a pivotal role in managing these deformities. The success of osteotomies in correcting lower-limb deformities and preserving joint function is highly dependent on meticulous preoperative planning. The mechanical axis of the limb, commonly referred to as the Mikulicz line, is employed to categorize the type of deformity. A lateral deviation of the Mikulicz line signifies a valgus deformity, whereas a medial deviation indicates a varus deformity. Furthermore, a precise assessment of the deformity’s location is essential, as abnormalities may arise from isolated or combined deviations within the lower limb. Complex deformities necessitate comprehensive measurements to facilitate accurate surgical planning [11].

Rapid advancements in automated radiological image processing software devices are observed. One study including 284 patients compared the artificial intelligence (AI) outputs to those manually measured across alignment assessments in lower extremities using radiographs. Interestingly, automated measurements of knee alignment and length measurements produced with AI tools result in measures that are reproducible and accurate compared to those acquired manually [12].

### 1.5. Rotation Deformities

**Figure 3:**
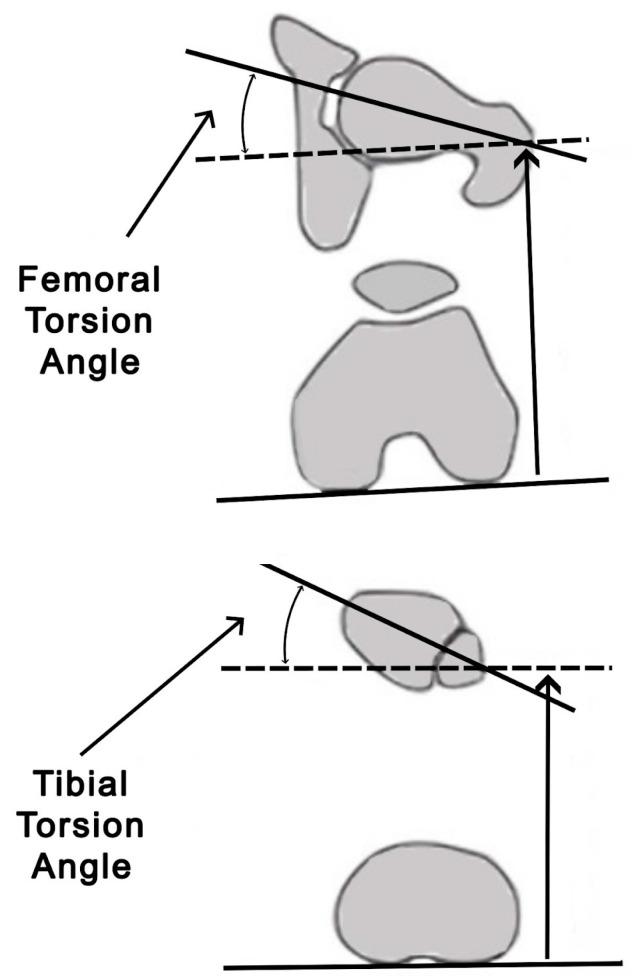
Rotation deformities: femoral torsion angle and tibial torsion angle (based on Luís et al. [8]).

Torsion refers to the rotational displacement of bone segments along the longitudinal axes of the long bones. Rotation deformities might arise from congenital conditions affecting the acetabulum, femur, and tibia, or from growth-related disorders. Additionally, they can result from post-traumatic incidents. These deformities can lead to complications including joint arthrosis, patellar instability, and the degeneration of the patellofemoral cartilage [13]. Lower-limb rotation deformities can be assessed clinically and radiographically. CT is the preferred method for the precise assessment of femoral and tibial rotation. CT imaging allows for the accurate measurement of angles between the distal and proximal joint axes in transverse planes (Figure 3) [14,15].

Consequently, in certain cases, it is advantageous to conduct additional diagnostic procedures to accurately identify and evaluate the specific issues and underlying conditions associated with lower-limb alignment deformities [16].

## 2. Imaging Methods

Imaging modalities assessing lower-limb alignment exhibit certain limitations.

Although radiographs represent the most available and cost-effective method, they require proper patient positioning to ensure accurate results. Coronal and sagittal alignments of lower extremities are primarily assessed using full-length weight-bearing AP radiographs, which are standard two-dimensional (2D) imaging techniques. However, it has been noted that measurements derived from 2D imaging might be affected by knee flexion and axial plane rotation [17]. It is noteworthy that a significant proportion of patients with osteoarthritic knees present with flexion contractures and rotational deformities. This deviations could adversely affect the evaluation of coronal alignment [18,19,20].

To address the limitations of two-dimensional imaging in evaluating lower-extremity alignment, three-dimensional (3D) techniques such as CT, MRI, and intraoperative navigation systems could be employed [21,22,23]. These advanced methods enhance the accuracy of alignment assessments, thereby improving diagnostic and treatment strategies. Nonetheless, these techniques do not accurately represent the true weight-bearing conditions which may introduce potential inaccuracies in the evaluations and raise questions about their cost-effectiveness [24,25,26]. Within-person mechanical leg axis measurements were found to be different when comparing weight-bearing full-length radiographs to non-weight-bearing measurement modalities. The discrepancy between measurement modalities may arise from a real difference in alignment between supine and the weight-bearing status of the patient, which has been recognized in prior research [27].

With the CT method, the reliability and reproducibility of alignment assessments are compromised by positional variables that impact the identification of the anatomical axis and the accuracy of measurements. Additionally, this method is associated with higher exposure to ionizing radiation [28]. Tarassoli et al. demonstrated the superiority of using CT over full-length standing radiographs due to an underestimation of proximal tibial varus using radiographs [29]. Other study showed that measurements derived from three-dimensional models (3D models) based on upright biplanar linear radiographs enable assessments of lower-limb length and alignment angles that are comparable to those obtained from supine CT scans and full-length radiographs [30].

A meta-analysis evaluating the accuracy of patient-specific cutting guides generated from MRI versus CT imaging methods in TKA demonstrated the superiority of MRI due to the lower proportion of coronal plane outliers with regard to overall limb mechanical axes. However, the analysis revealed no significant differences between MRI and CT regarding the sagittal placement of femoral and tibial components, as well as the coronal placement of these components and the axial rotation of the femoral component [31]. In a study consisting of 45 patients, the results of preoperative standing full-length alignment radiographs were compared with supine MRI assessments of lower-limb alignment prior to TKA. The findings from this study indicate that supine MRI underestimates the degree of deformity at the knee joint [32].

Further research in a larger patient population is essential in order to systematically compare the available imaging modalities for preoperative assessment, in turn allowing us to identify a method that demonstrates the greatest efficacy in evaluating deformities.

## 3. Discussion

Lower-limb deformities present a diverse spectrum of clinical and radiological manifestations, ranging from angular misalignments and rotational abnormalities to limb-length discrepancies. These deformities, whether congenital, developmental, or acquired, can significantly impact a patient’s mobility, function, and quality of life. The complexity and variability of these conditions demand meticulous preparation and strategic planning from the orthopedic surgeon to ensure optimal corrective outcomes. A thorough understanding of the underlying pathology, precise assessment of the deformity, and careful consideration of both surgical and non-surgical interventions are paramount. Therefore, a structured and systematic approach to preoperative planning is essential. This involves comprehensive patient evaluation, detailed imaging analysis, and the use of appropriate planning tools to tailor the surgical approach to the specific deformity. By providing a clear and standardized framework for preoperative planning, surgeons can minimize complications, enhance surgical precision, and improve long-term functional outcomes for patients with lower-limb deformities [33].

This study focused on evaluating lower-limb alignment on full-length standing radiographs in the AP projection and rotational deformities in the transverse plane. Although the evaluation of lower-limb alignment in the frontal plane is well established in musculoskeletal research, particularly in the cases of knee osteoarthritis [34], there are some knowledge gaps in the literature related to radiographic methods for the assessment of the alignment in the sagittal plane. The visualization of anatomical structures needed to assess lower-limb deformations in the sagittal plane is challenging due to differences in the intensity of the X-ray beams required to visualize both the femoral head and the knee [35]. Additionally, discrepancies in the standardization of angular measurements for sagittal plane assessment further complicate this issue [36]. Indeed, in clinical practice, the assessment of sagittal plane deformities is less common than that of frontal plane deformities. The mentioned reasons may explain the limitations of research using radiography to assess lower-limb alignment in the sagittal plane. Nevertheless, given its clinical significance, the development of future research utilizing the sagittal plane for lower-limb deformities is anticipated.

Variations in lower-limb alignment appear to be influenced by many factors, even ethnic origin, as demonstrated by differences in specific alignment parameters across populations. For example, Japanese individuals have been found to exhibit smaller mechanical, lateral, distal, femoral, and medial proximal tibial angles, as well as a larger HKA angle and a higher percentage of constitutional varus compared to individuals from other countries. These findings highlight the importance of considering population-specific anatomical characteristics when planning lower-limb surgeries, such as total knee arthroplasty [37].

Significant advancements in automated radiological image processing, particularly AI-driven tools, have led to highly accurate and reproducible measurements of knee alignments and limb lengths. Current advanced techniques are available for the precise measurement of lower-limb alignment, outperforming conventional two-dimensional (2D) standing radiographs. The introduction of automatic calculation methods using three-dimensional (3D) bone models derived from CT scans enables more efficient preoperative planning for orthopedic surgeries. These methods allow for a comprehensive quantification of lower-limb alignment parameters with significantly reduced times compared to manual assessments. Moreover, the accuracy of these automatic methods is comparable to, or even exceeds, the consistency of repeated manual measurements, making them a valuable and reliable alternative for precise alignment evaluation [38].

There is a necessity for further data to optimize the application of AI methods in the radiological assessment of lower-limb deformities. Research should focus on the development of diverse and high-quality imaging datasets, and it should also represent various ethnic groups, which will significantly enhance the accuracy of AI algorithms in automated assessments. It is essential to evaluate the performance of AI systems across different populations to minimize potential biases. Establishing standardized protocols for clinical validation, alongside the integration of AI tools into radiological workflows, will be critical for achieving precise real-time measurements and facilitating the effective implementation of AI-based systems in clinical practice.

## 4. Conclusions

Lower-limb alignment deformities present significant clinical challenges, potentially leading to severe complications, such as progressive degenerative diseases and disabilities. Full-length standing radiographs in AP projection are useful for obtaining accurate measurements of the anatomical and mechanical axes, angles, and deviations of the lower-limb. These measurements are crucial for effective preoperative planning and the successful correction of deformities through osteotomy and TKA. Alternative modalities like CT or MRI are crucial for precise assessment in more complicated cases. There are promising prospects for the implementation of AI-based automated measurement systems. Moreover, there are promising prospects for the implementation of AI-based automated measurement systems. There is a necessity to systematically compare the available imaging modalities for preoperative lower-limb alignment assessments and to further validate research on artificial intelligence methods to enable their application in routine clinical practice.

## Figures and Tables

**Table 1:** The physiological range for mechanical lower-limb angles.

Mechanical Angle	Physiological Ranges
Mechanical lateral proximal femoral angle (mLPFA)	90 ± 5°
Mechanical lateral distal femoral angle (mLDFA)	87 ± 3°
Mechanical medial proximal tibial angle (mMPTA)	87 ± 3°
Mechanical lateral distal tibial angle (mLDTA)	89 ± 3°

## Data Availability

No new data were created or analyzed in this study.
